# Supplementary material for: The anti-cancer drugs curaxins target spatial genome organization
Source: Nat Commun. 2019 Mar 29;10:1441. doi: 10.1038/s41467-019-09500-7 (PMC6441033; doi:10.1038/s41467-019-09500-7)
Supplement: Supplementary file 1 — Supplementary Information [file 41467_2019_9500_MOESM1_ESM.pdf]

Supplementary Information

Anti-cancer drugs curaxins target the 3D genome organization

Kantidze et al.

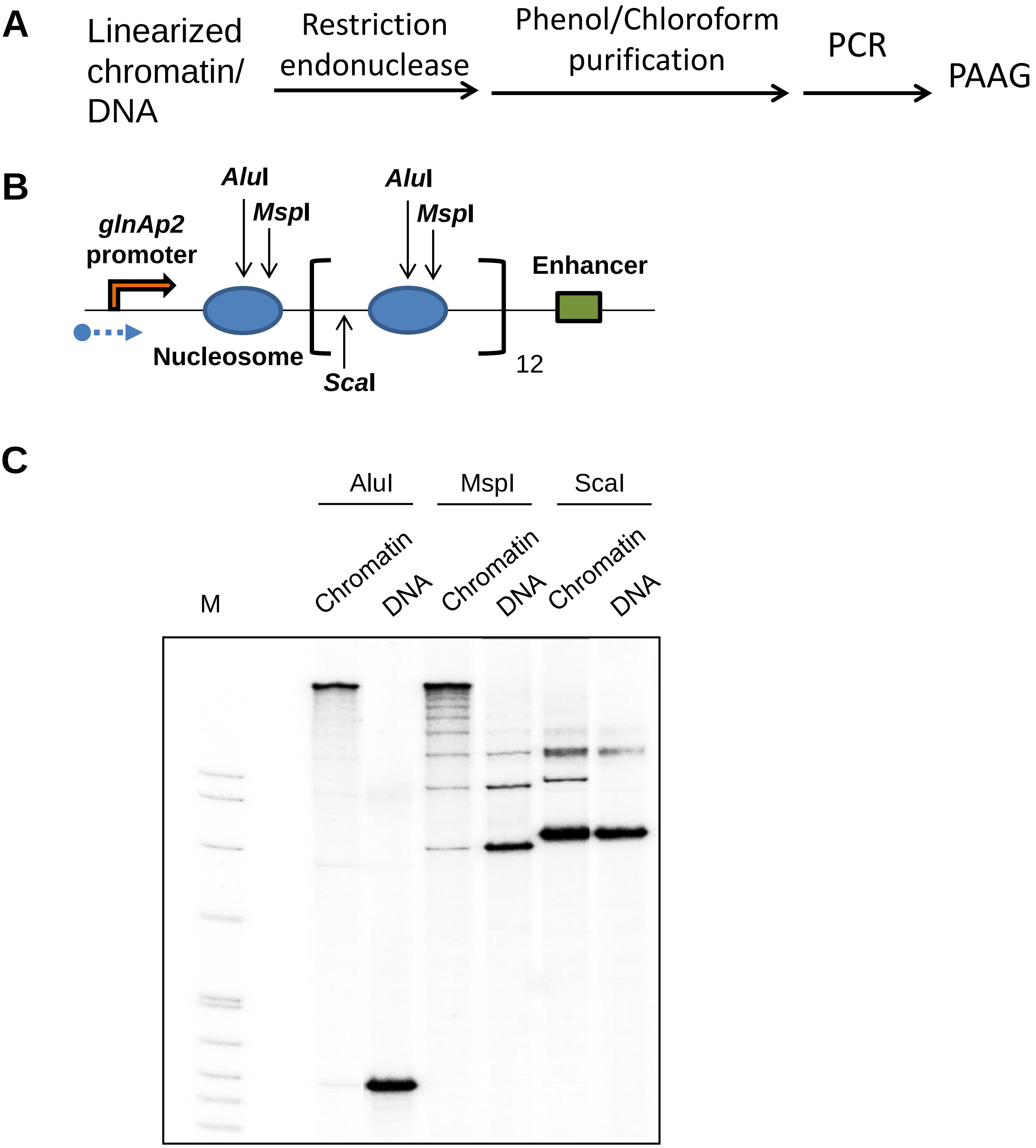

Supplementary Figure 1. Chromatin array analysis. **a** Experimental approach: a restriction enzyme sensitivity assay. $601_{207\times13}$, nucleosomal arrays were assembled on linearized plasmids and incubated in the presence of an excess of one of the following restriction enzymes *Alu*I, *Msp*I or *Sca*I. Purified DNA was subjected to primer extension followed by denaturing PAGE. **b** A Scheme of restriction enzymes digestion: *Alu*I cut on the middle of 601 sequence, *Msp*I cut on the site covered by H2A/H2B dimer, *Sca*I cut sequence between nucleosomes. Blue dashed arrow – primer for extension by PCR. **c** Analysis of end-labeled DNA by denaturing PAGE. Note that chromatin assembly results in almost quantitative protection of the templates from *Alu*I and *Msp*I. M: pBR322-*Msp*I digest.

A

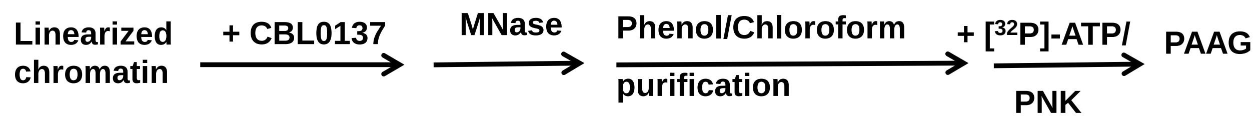

B

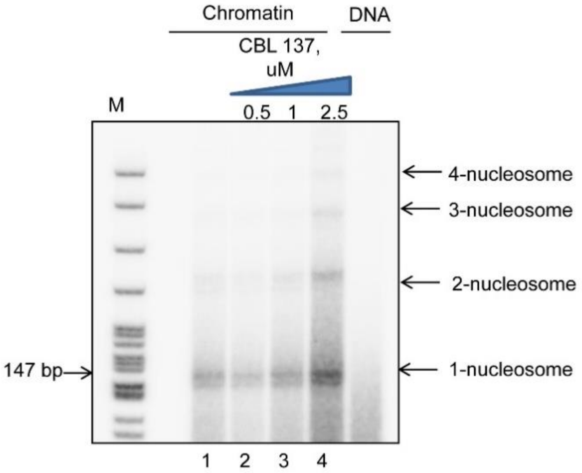

C

Lane number	1	2	3	4
4-Nucleosome, %	1.3	1.3	1.6	1.2
3-Nucleosome, %	3.1	3.1	3.5	3.6
2-Nucleosome, %	11.6	12.3	13.7	14.2
1-Nucleosome, %	84.1	83.2	81.3	81

Supplementary Figure 2. CBL0137 minimally affects nucleosome structure. **a** Experimental approach: the chromatin arrays were incubated at increasing concentrations of CBL0137 (0.5, 1 and 2.5 uM) and were subject to micrococcal nuclease (MNase) digestion. DNA was purified and labeled by PNK/[^{32}P]-ATP. **b** Analysis of purified DNA by PAGE. M: pBR322-*Msp*I digest. **c** The gel shown in **b** was quantified using a PhosphorImager. Amount of label present in each band was calculated as % of label present in all four bands (1-nucleosome etc.).

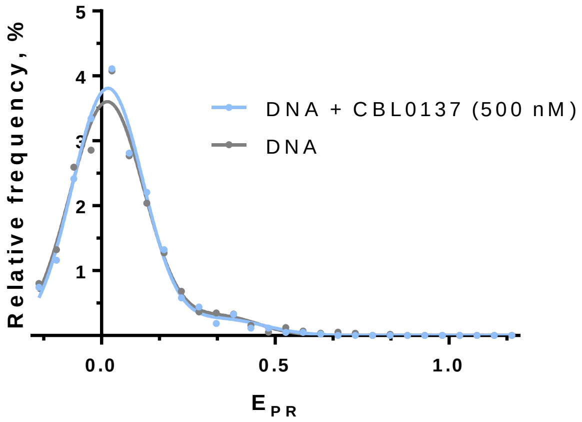

Supplementary Figure 3. spFRET microscopy of histone-free fluorescently labeled DNA in the absence or presence of curaxin CBL0137. Typical frequency distributions of free DNA by proximity ratio (E_{PR}) are shown. The experimental data (dots) were fitted with as a sum of two Gaussians (solid lines). The sample sizes (n, single particle events) were the following: (DNA) – 1176; (DNA+CBL0137) – 880. The mean values of the main E_{PR} peak maxima and S.E.M. averaged over 3 independent experiments were the following: (DNA) – 0.02±0.01; (DNA+CBL0137) – 0.02±0.02.

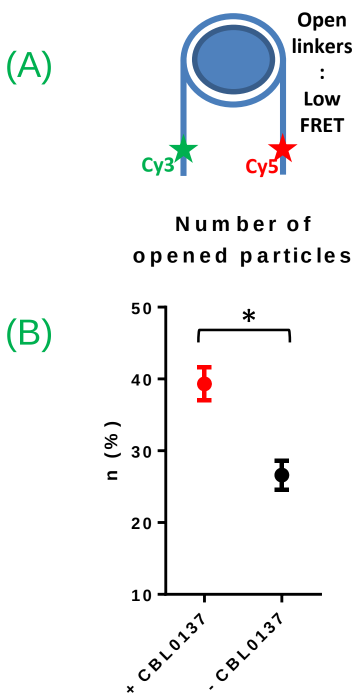

Supplementary Figure 4. Quantitation of open-linker (low FRET) nucleosomes in presence and absence of CBL0137. **a** Experimental approach. The mononucleosomes containing the single pair of Cy3 and Cy5 dyes on the nucleosomal linker DNA (the positions of the dyes are shown by green and red asterisks, respectively). spFRET from nucleosomes was measured in the absence or presence of curaxin CBL0137. **b** Comparison of the relative amount (n) of nucleosomes with opened linkers in presence and absence of CBL0137. Mean values and standard deviations are shown (n=3). Asterisk – according to unpaired t-test the two-tailed p value is equal to 0.015, indicating that the analyzed values are significantly different ($p<0.05$).

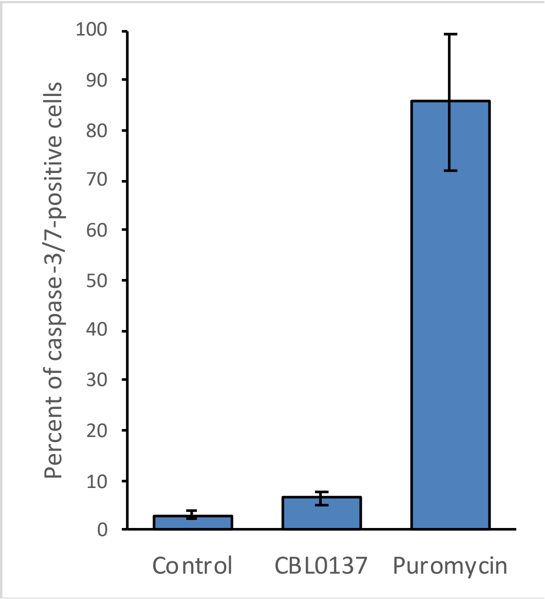

Supplementary Figure 5. Treatment of HT1080 cells with CBL0137 (3 uM, 6 h) did not lead to meaningful apoptosis. Percent of caspase-3/7-positive cells is shown in each case. HT1080 cells treated with puromycin (1 μg/ml, 12 h) were used as a positive control. Results of five independent experiments are shown.

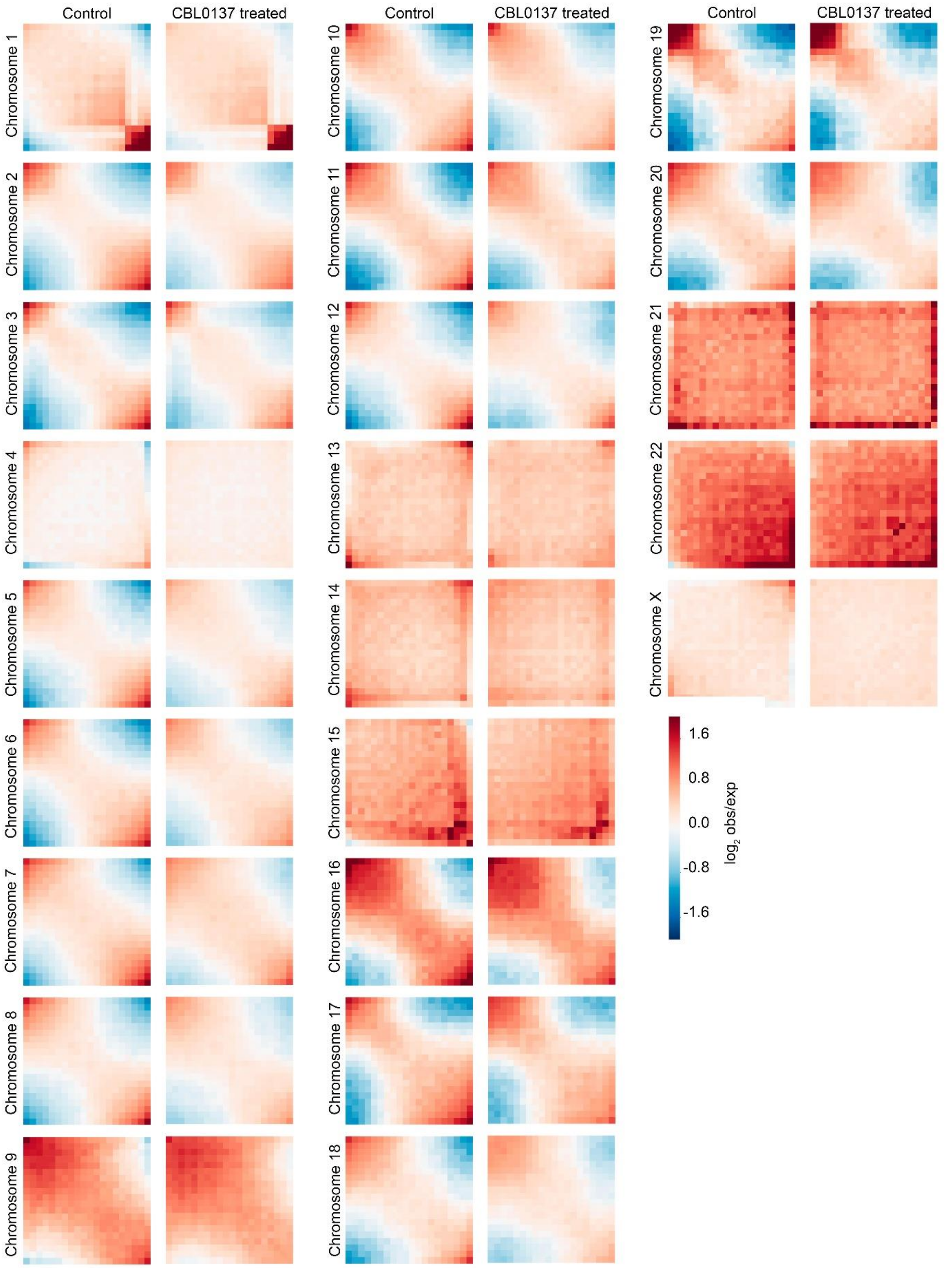

Supplementary Figure 6. Saddle plots displaying the extent of compartmentalization of individual chromosomes in control and CBL0137-treated cells.

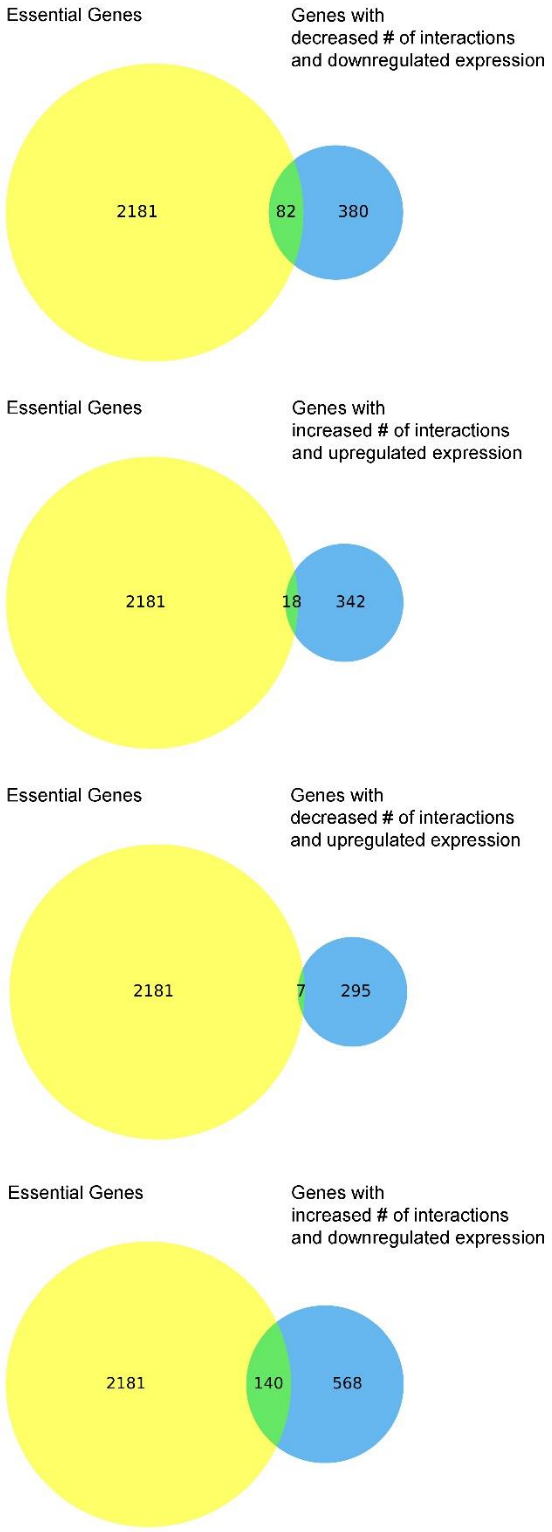

Supplementary Figure 7. Venn diagrams showing the overlaps between so-called "essential genes" (Blomen et al. Science 2015) and genes with changed contact profiles upon CBL0137-treatment of HT1080 cells.

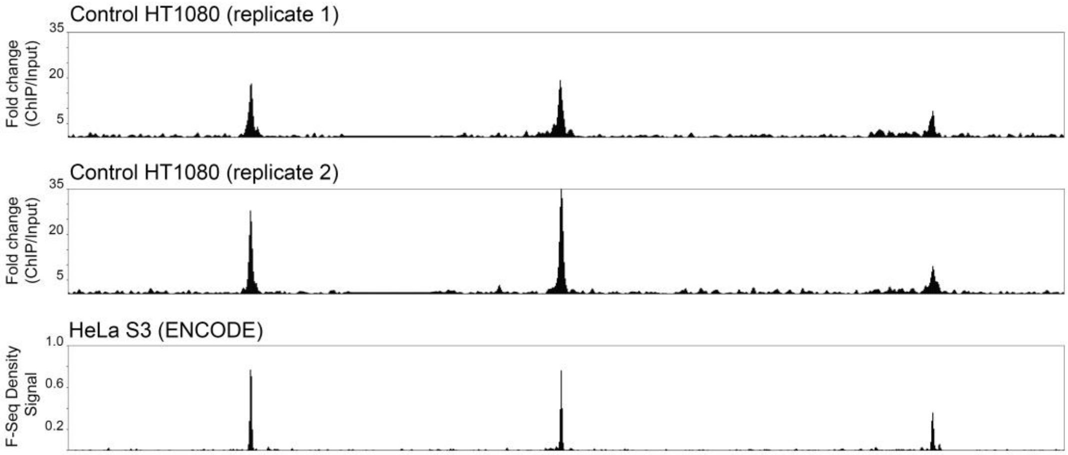

Supplementary Figure 8. CTCF distribution across the particular genomic region (chr6:135,455,856-135,512,644) analyzed by ChIP-sequencing. Histograms present data for intact HT1080 cells (obtained in our study; results of two biological replicates are shown) or HeLa S3 cells (data was extracted from ENCODE).

anti-MYC

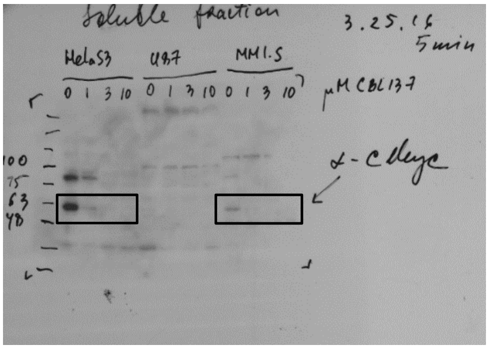

anti-MYC

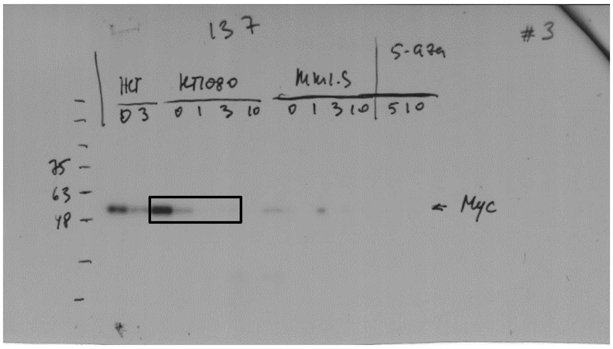

anti-Actin

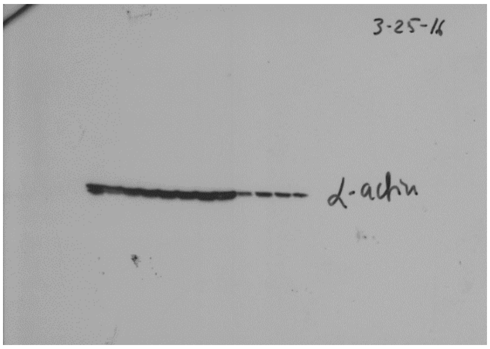

anti-Actin

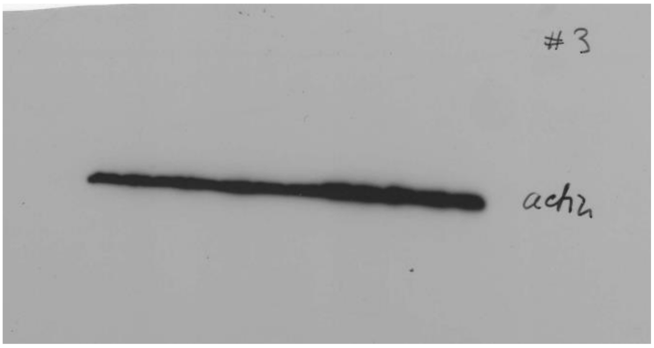

Supplementary Figure 9. Full size, uncropped immunoblots for Figure 1.

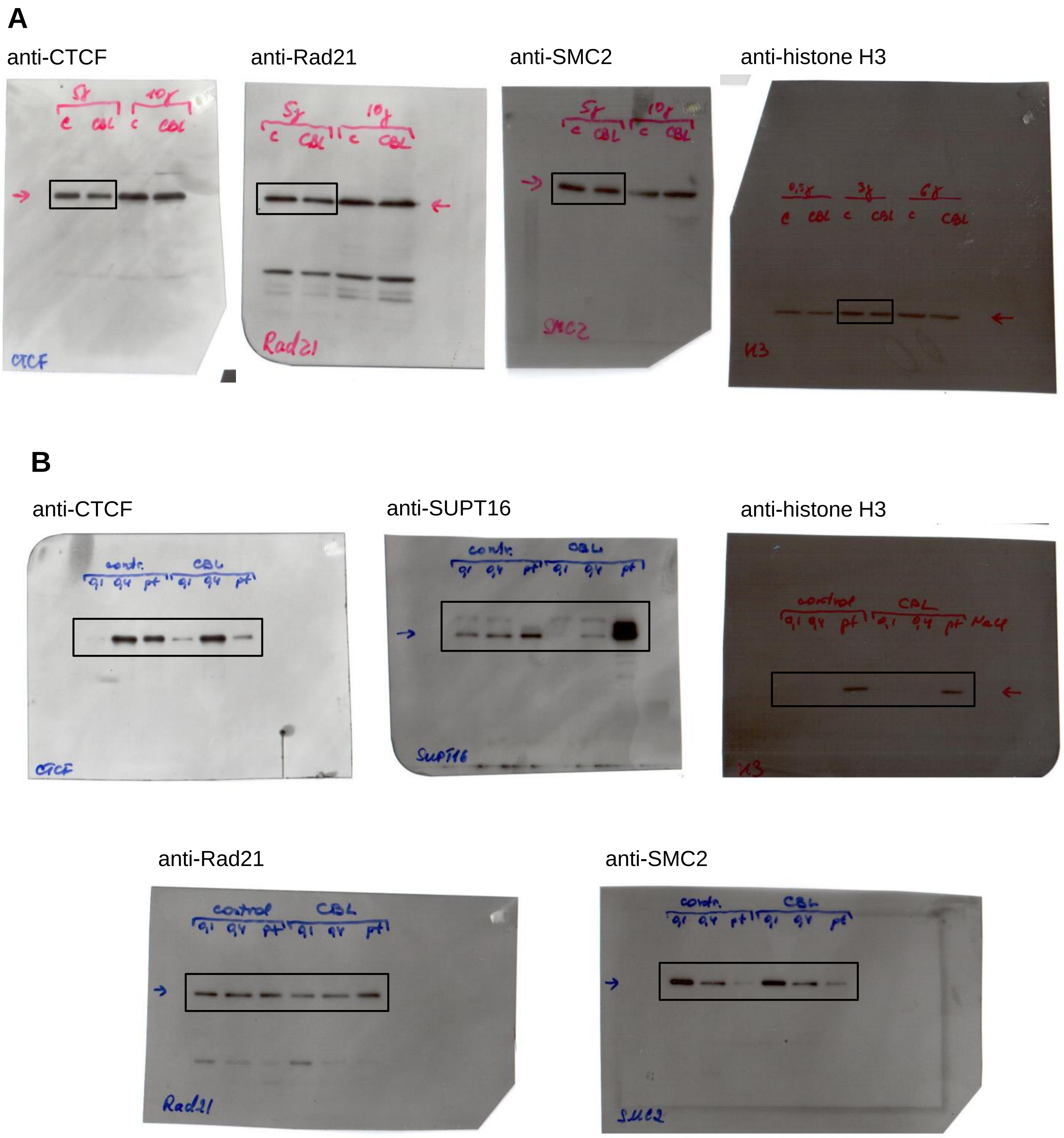

Supplementary Figure 10. Full size, uncropped immunoblots for Figures 6a (A) and 6b (B).
