# Supplementary material for: The anti-cancer drugs curaxins target spatial genome organization
Source: Nat Commun. 2019 Mar 29;10:1441. doi: 10.1038/s41467-019-09500-7 (PMC6441033; doi:10.1038/s41467-019-09500-7)
Supplement: Supplementary file 3 — Description of Additional Supplementary Files [file 41467_2019_9500_MOESM3_ESM.pdf]

Description of Additional Supplementary Files

File Name: Supplementary Data 1
Description: A list of PSYHIC-annotated spatial contacts between genes and potential remote regulatory elements.

File Name: Supplementary Data 2
Description: Statistics of the Hi-C libraries sequencing and mapping, and the results of specialized Hi-C reproducibility tests.
