# Supplementary material for: The anti-cancer drugs curaxins target spatial genome organization
Source: Nat Commun. 2019 Mar 29;10:1441. doi: 10.1038/s41467-019-09500-7 (PMC6441033; doi:10.1038/s41467-019-09500-7)
Supplement: Supplementary file 6 — Reporting Summary [file 41467_2019_9500_MOESM6_ESM.pdf]

natureresearch

Corresponding author(s): Sergey V. Razin, Vasily M. Studitsky, Katerina V. Gurova

Last updated by author(s): Feb 7, 2019

Reporting Summary

Nature Research wishes to improve the reproducibility of the work that we publish. This form provides structure for consistency and transparency in reporting. For further information on Nature Research policies, see Authors & Referees and the Editorial Policy Checklist.

Statistics

For all statistical analyses, confirm that the following items are present in the figure legend, table legend, main text, or Methods section.

n/a	Confirmed	
☐	☒	The exact sample size (n) for each experimental group/condition, given as a discrete number and unit of measurement
☐	☒	A statement on whether measurements were taken from distinct samples or whether the same sample was measured repeatedly
☐	☒	The statistical test(s) used AND whether they are one- or two-sided<br>*Only common tests should be described solely by name; describe more complex techniques in the Methods section.*
☒	☐	A description of all covariates tested
☒	☐	A description of any assumptions or corrections, such as tests of normality and adjustment for multiple comparisons
☒	☐	A full description of the statistical parameters including central tendency (e.g. means) or other basic estimates (e.g. regression coefficient) AND variation (e.g. standard deviation) or associated estimates of uncertainty (e.g. confidence intervals)
☒	☐	For null hypothesis testing, the test statistic (e.g. F, t, r) with confidence intervals, effect sizes, degrees of freedom and P value noted<br>*Give P values as exact values whenever suitable.*
☒	☐	For Bayesian analysis, information on the choice of priors and Markov chain Monte Carlo settings
☒	☐	For hierarchical and complex designs, identification of the appropriate level for tests and full reporting of outcomes
☐	☒	Estimates of effect sizes (e.g. Cohen's d, Pearson's r), indicating how they were calculated

Our web collection on statistics for biologists contains articles on many of the points above.

Software and code

Policy information about availability of computer code

Data collection	n/a
Data analysis	FastQC v11.0, Bowtie2 (version 2.2.3), Samtools (version 1.5), PePr (version 1.1.24), Bedtools (version 2.27.1), deepTools(version 2.0), pybedtools package, pyBigWig package, hiclib, cooler (version 0.9.7), Lavaburst package, PSYCHIC, QuASAR-QC, QuASAR-Rep, HiC-Spector, GenomeDISCO.

For manuscripts utilizing custom algorithms or software that are central to the research but not yet described in published literature, software must be made available to editors/reviewers. We strongly encourage code deposition in a community repository (e.g. GitHub). See the Nature Research guidelines for submitting code & software for further information.

Data

Policy information about availability of data

All manuscripts must include a data availability statement. This statement should provide the following information, where applicable:

- Accession codes, unique identifiers, or web links for publicly available datasets
- A list of figures that have associated raw data
- A description of any restrictions on data availability

All datasets reported in this paper are available at the Gene Expression Omnibus with accession numbers GEO: GSE122463, GSE117611, GSE117409, GSE107633. The source data underlying Figs 1a-e, 2c-f, 3c-e, 5a, 5c, and 6a-b and Supplementary Figures 3, 4, and 5 are provided as a Source Data file.

Field-specific reporting

Please select the one below that is the best fit for your research. If you are not sure, read the appropriate sections before making your selection.

☒ Life sciences ☐ Behavioural & social sciences ☐ Ecological, evolutionary & environmental sciences

For a reference copy of the document with all sections, see nature.com/documents/nr-reporting-summary-flat.pdf

Life sciences study design

All studies must disclose on these points even when the disclosure is negative.

Sample size	No sample-size calculation was performed.
Data exclusions	No data were excluded from the analysis.
Replication	Spearman correlation coefficient analysis showed high reproducibility of ChIP-seq experiments (0.996 for control replicates and 0.991 for CBL0137-treated replicates). Reproducibility of Hi-C experiments was analyzed by specialized software (GenomeDISCO, HiC-Spector), that also showed high reproducibility between replicates (full data are presented in the Supplementary Table 1).
Randomization	n/a
Blinding	n/a

Reporting for specific materials, systems and methods

We require information from authors about some types of materials, experimental systems and methods used in many studies. Here, indicate whether each material, system or method listed is relevant to your study. If you are not sure if a list item applies to your research, read the appropriate section before selecting a response.

Materials & experimental systems

n/a	Involved in the study	
☐	☒	Antibodies
☐	☒	Eukaryotic cell lines
☒	☐	Palaeontology
☒	☐	Animals and other organisms
☒	☐	Human research participants
☒	☐	Clinical data

Methods

n/a	Involved in the study	
☐	☒	ChIP-seq
☒	☐	Flow cytometry
☒	☐	MRI-based neuroimaging

Antibodies

Antibodies used	c-MYC (clone 9E10, Santa Cruz Biotechnology, cat. #sc-40), beta-actin (Sigma-Aldrich, cat. # A3854); CTCF (Active Motif, cat. #61311); Rad21 (Abcam, cat. #ab992); SMC2 (Cell Signalling, cat. #5394); SPT16 (Abnova, cat. #MAB8018); histone H3 (Abcam, cat. #ab1791).
Validation	Specificity of all antibodies was validated by manufacturers and Western blot analysis in this study.

Eukaryotic cell lines

Policy information about cell lines

Cell line source(s)	HeLa and MM1.S cells were obtained from ATCC. HT1080 cells were obtained from Andrei Gudkov lab (Roswell Park Cancer Institute) and were authenticated by short tandem repeat PCR to be 100% identical to HT1080 cells from ATCC (ATCC® CCL-121™).
Authentication	HeLa and MM1.S cells were obtained from ATCC.<br>HT1080 cells were were authenticated by short tandem repeat PCR to be 100% identical to HT1080 cells from ATCC (ATCC® CCL-121™).
Mycoplasma contamination	All cell lines were tested and showed no contamination with mycoplasma.
Commonly misidentified lines (See ICLAC register)	Cell lines used in the study are not listed in the database of commonly misidentified cell lines, ICLAC (http://iclac.org/databases/cross-contaminations),

ChIP-seq

Data deposition

☒ Confirm that both raw and final processed data have been deposited in a public database such as GEO.

☒ Confirm that you have deposited or provided access to graph files (e.g. BED files) for the called peaks.

Data access links<br>*May remain private before publication.*	https://www.ncbi.nlm.nih.gov/geo/query/acc.cgi?acc=GSE122463
Files in database submission	CTCF ChIP Control HT1080 cells – 2 biological replicates, CTCF ChIP CBL0137-treated HT1080 cells - 2 biological replicates, CTCF Input Control HT1080 cells – 2 biological replicates, CTCF Input CBL0137-treated HT1080 cells - 2 biological replicates.
Genome browser session<br>(e.g. UCSC)	no longer applicable

Methodology

Replicates	CTCF ChIP Control HT1080 cells – 2 biological replicates, CTCF ChIP CBL0137-treated HT1080 cells - 2 biological replicates, CTCF Input Control HT1080 cells – 2 biological replicates, CTCF Input CBL0137-treated HT1080 cells - 2 biological replicates.
Sequencing depth	ChIP Control replicate 1 – total = 58883351, uniquely mapped=39183608, read length=75bp, single-end,<br>ChIP Control replicate 2 – total =59474535, uniquely mapped=40456002, read length=75bp, single-end,<br>ChIP CBL0137 replicate 1 – total = 56399178, uniquely mapped=38448319, read length=75bp, single-end,<br>ChIP CBL0137 replicate 2 – total =60277417, uniquely mapped=40812365, read length=75bp, single-end,<br>Input Control replicate 1 – total =60533468, uniquely mapped=44058191, read length=75bp, single-end,<br>Input Control replicate 2 – total =61845165, uniquely mapped=45126135, read length=75bp, single-end,<br>Input CBL0137 replicate 1 – total =59682212, uniquely mapped=43432522, read length=75bp, single-end,<br>Input CBL0137 replicate 2 – total =52581826, uniquely mapped=38363337, read length=75bp, single-end
Antibodies	ActiveMotif anti-CTCF pAb, Catalog No: 61311, Lot#: 34614003
Peak calling parameters	Reads were mapped using bowtie2 (version 2.2.3) – very-sensitive option. Non-uniquely mapped reads were filtered using 'XS:i' flag. Resulted sam files were sorted,possible PCR and optical duplicates were filtered using Samtools (version 1.5). Peaks were called using PePr (https://github.com/shawnzhangyx/PePr, PMID: 24894502) with p-value cutoff 0.05 and sliding window size 100bp.
Data quality	Raw reads quality control was performed used FastQC v11.0. Number of peaks in Control with FDR 5% and above 5-fold enrichment =47459, number of peaks in CBL0137-treated with FDR 5% and above 5-fold enrichment = 29544.
Software	FastQC v11.0, Bowtie2 (version 2.2.3), Samtools (version 1.5), PePr (version 1.1.24), Bedtools v2.27.1
